# Supplementary material for: COVID-19 Vaccination in Multiple Sclerosis and Inflammatory Diseases: Effects from Disease-Modifying Therapy, Long-Term Seroprevalence and Breakthrough Infections
Source: Vaccines (Basel). 2022 Apr 28;10(5):695. doi: 10.3390/vaccines10050695 (PMC9146267; doi:10.3390/vaccines10050695)
Supplement: Supplementary file 1 [file vaccines-10-00695-s001.zip › vaccines-1673747-supplementary.pdf]

Table S1. Absolute IgG index between different DMTs and vaccines used.

DMT – IgG index	BNT162b2	mRNA-1273	Ad26.COV2.S	p-value
No DMT	n = 42<br>16.1 (6.7)	n = 42<br>16.4 (6.2)	n = 6<br>2.3 (1.4)	**<0.001**
IFN-β	n = 32<br>15.7 (6.4)	n = 35<br>17.3 (5.1)	n = 6<br>6.7 (7.1)	**<0.001**
GA	n = 25<br>15.6 (7.1)	n = 25<br>19.1 (3.0)	n = 2<br>2.7 (0.1)	**<0.001**
Fumarate	n = 25<br>14.0 (7.9)	n = 19<br>17.9 (4.6)	-	**0.023**
Teriflunomide	n = 11<br>15.3 (7.1)	n = 9<br>14.3 (8.6)	n = 1<br>1.0	0.146
S1Ps	n = 5<br>2.8 (2.1)	n = 1<br>20.0	-	0.131
Natalizumab	n = 28<br>16.5 (6.2)	n = 20<br>17.5 (6.0)	n = 5<br>10.7 (8.6)	0.179
Cladribine	n = 3<br>8.2 (10.3)	n = 3<br>12.3 (6.6)	-	0.591
Off-label DMT	n = 8<br>13.7 (7.4)	n = 7<br>13.0 (8.8)	-	0.675
Anti-CD20 mAb	n = 11<br>13.7 (8.7)	n = 18<br>9.4 (8.2)	n = 1<br>1.2	0.298
Alemtuzumab	n = 4<br>20.0	n = 1<br>3.3	-	-

Legend: MS – multiple sclerosis, DMT – disease modifying therapy, IFN – interferon, S1Ps – sphingosine-1-phosphate, SD – standard deviation, mAb – monoclonal antibody. Statistically significant p-values are shown in bold.

Rituximab, ocrelizumab and ofatumumab were all grouped under the category of Anti-CD20 mAb. Fingolimod, ponesimod, siponimod and ozanimod were grouped under the S1P category. Both dimethyl fumarate and diroximel fumarate were grouped under Fumarates. Off-label MS medications included, cyclophosphamide, intravenous immunoglobulin, azathioprine, methotrexate and mycophenolic acid.

The maximal reported IgG index is at 20.0.

One-way analysis of variance (ANOVA) was used. P-values lower than 0.05 were considered statistically significant and shown in blod.

Table S2. Changes in absolute IgG index over 6 months after full vaccination course.

Vaccine	Timepoint	Median (IQR)	Number of samples	p-value
BNT162b2	4-weeks	20.0.1 (1.0-20.0)	49	**0.004**
	6-months	6.7 (0.0-9.9)	49	
mRNA-1273	4-weeks	20.0(20.0-20.0)	41	**0.014**
	6-months	10.4 (3.4-20.0)	41	
Ad26.COV2.S	4-weeks	1.6 (0.9-4.8)	10	0.161
	6-months	0.0 (0.0-2.2)	10	

Legend: IQR – interquartile range, P-values lower than 0.05 were considered statistically significant and shown in bold. The maximal reported IgG index is at 20.0. P-values lower than 0.05 were considered statistically significant and shown in blod.
